# The Effects of *Cannabis sativa* L. Extract on Oxidative Stress Markers In Vivo

**DOI:** 10.3390/life11070647

**Published:** 2021-07-02

**Authors:** Asta Kubiliene, Karolina Mickute, Juste Baranauskaite, Mindaugas Marksa, Arunas Liekis, Ilona Sadauskiene

**Affiliations:** 1 Department of Analytical and Toxicological Chemistry, Faculty of Pharmacy, Medical Academy, Lithuanian University of Health Sciences, Sukileliu St. 13, LT-50161 Kaunas, Lithuania; karolinamickute10@gmail.com (K.M.); juste.baranauskaite@lsmuni.lt (J.B.); mindaugas.marksa@lsmuni.lt (M.M.); 2 Neuroscience Institute, Lithuanian University of Health Sciences, Eiveniu St. 4, LT-50161 Kaunas, Lithuania; arunas.liekis@lsmuni.lt (A.L.); ilona.sadauskiene@lsmuni.lt (I.S.)

**Keywords:** *Cannabis*, HPLC, antioxidant activity, mice

## Abstract

In recent decades, a lot of attention has been paid to *Cannabis sativa* L. due to its useful applications, including in fibers, oil, food for humans and animals, and therapeutics. The present study aimed to determine antioxidant activity of cannabinoids in *Cannabis sativa* L. in vivo, evaluating the possible antioxidative effect of *Cannabis sativa* L. extract (CE) on malondialdehyde (MDA) and glutathione (GSH) concentrations as well as on catalase (CAT) activity in BALB/c mice. In total, 40 mice were divided into five equal groups: the aluminum group (7.5 mg AlCl_3_/kg/d (0.15 LD_50_), the saline group, the 10% ethanol group (an appropriate amount of the solution for mouse weight), the CE group (1.6 mg CE/g/day), and the aluminum-CE group (7.5 mg AlCl_3_ plus 1.6 mg CE/g/day). The results of the study showed that CE significantly decreased (by 26.81%, *p* < 0.05) the concentration of GSH in blood of the mice and the concentration of MDA in the brain (by 82.12%) and liver (by 53.5%) of the mice compared to the respective concentrations in the AlCl_3_ group. CE significantly (*p* < 0.05) increased CAT activity in the brain (by 64.79%) and liver (by 72.37%) of the mice after the AlCl_3_-induced prooxidant effect. The results showed the antioxidant activity of cannabidiolic acid (CBDA) in vitro. The findings in vivo indicate that *Cannabis sativa* L. is a good source of natural antioxidants and can be used in the management of oxidative stress.

## 1. Introduction

Oxidative stress resulting from the formation of free radicals plays an important role in the development of various diseases, such as cancer, rheumatoid arthritis, atherosclerosis, diabetes, cardiovascular diseases, myocardial infarction, post-ischemic perfusion injury, chronic inflammation, and some degenerative diseases in human [1]. Free radicals are widespread in nature, aluminum (Al^3+)^ being one of the most widely spread elements. It is released naturally into aquatic ecosystems as a result of rock or soil erosion. However, in recent years, environmental levels of Al^3+^ have increased due to diverse anthropogenic activities. Although Al^3+^ is considered innocuous, this metalloid is capable of causing oxidative stress and the formation of reactive oxygen species (ROS) [2]. Aluminum can enter the human body via drinking water, beverages, food, or Al^3+^-containing drugs [3]. Exposure to high concentrations of aluminum may induce damage ranging from blood cell changes to brain, kidney, muscle, and bone pathologies [4]. An increased amount of Al^3+^ has been found in biological systems in the presence of pathological conditions [5]. In vitro studies have shown that Al^3+^ exposure can induce several biological effects implicated in the pathogenesis of Alzheimer’s disease, including apoptosis, mitochondrial gene expression, inflammation [6], cardiotoxicity [7], nephrotoxicity [8], hepatotoxicity [9], hematotoxicity [10], and bone and lung toxicity [7].

Antioxidants protect the body from the effects of free radicals, inhibit the oxidation of biomolecules, and prevent cell damage [11]. Recently, greater interest has been addressed to hemp (*Cannabis sativa* L.), also called industrial cannabis (the content of psychoactive cannabinoids <0.2%), which is mainly investigated due to the presence of more than 133 cannabinoids and terpenes in its composition [12]. Cannabinoids such as tetrahydrocannabinol (THC), cannabinol, and cannabidiol (CBD) have been found to be potent lipophilic antioxidants [13], and antioxidant activity of CBD and THC has been reported [14]. However, hemp produces other metabolites that could demonstrate antioxidant activity, as well [15]. Pollastro et al. reported that hemp synthesizes characteristic molecules, such as prenylated flavonoids, stilbenoid derivatives, and lignamides, which require further investigations [16]. Moreover, a special role is attributed to flavonoids acting as free-radical scavengers and chelators of metal ions. However, the hemp extracts were mainly chemically characterized and tested in vitro, while few data are available on their antioxidant role in bulk oil [12]. Therefore, the effect of the whole phyto-complex obtained from hemp inflorescences on lipid oxidation requires further in-depth investigation.

Nowadays, considerable attention has been directed towards the identification of natural (plant-derived) antioxidants that may be used for human consumption. *Cannabis sativa* L. is now gaining importance because of its potent antioxidant activity. Recent studies have shown that the active substances extracted from this plant have pharmacological properties such as insecticidal, antimicrobial, and antioxidant properties [17]. Hempseed has been shown to be a useful antioxidant [18]. Our recent study showed the ability of *Cannabis sativa* L. herb extracts to demonstrate antioxidant activity in vitro [19]. Still, the results of antioxidant properties in vitro and in vivo are not always the same. Consequently, the aim of this study was to determine antioxidant activity of cannabinoids in *Cannabis sativa* L. in vivo and to estimate the capability of *Cannabis sativa* L. extract to influence the levels of oxidative stress markers (the concentrations of GSH and MDA and CAT activity) in the blood, brain, and liver of experimental mice.

## 2. Materials and Methods

### 2.1. Materials

The hemp was collected from the experimental cultivation field of hemp *Cannabis sativa* L. in the North region of Lithuania. The aerial parts of the Futura 75 variety (THC ≤ 0.01%) were collected and deposited at the Herbarium of the Department of Analytical and Toxicological Chemistry at the Lithuanian University of Health Sciences. They were harvested in September 2018. After the drying process, the whole plant was stored at 4 °C prior to extraction. The moisture of the plant was 7.2 ± 0.5%.

### 2.2. Solvent and Reagents

Potassium chloride, phosphoric acid, and hydrogen peroxide were purchased from Merck (Darmstadt, Germany). Ethanol (96%) was supplied from Vilnius Degtine (Vilnius, Lithuania). Purified water was produced using a Millipore water purification system (Merck, Kenilworth, NJ, USA). Ammonium molybdate, ammonium formate ABTS (2,2-azinobis-(3-ethylbenzothiazoline-6-sulfonate), and DPPH (2,2-diphenyl-1-picryl-hydrazyl-hydrate) reagents were purchased from Sigma-Aldrich (Schnelldorf, Germany). Acetonitrile was purchased from Sigma-Aldrich (Buchs, Switzerland). Formic acid was purchased from Fluka Chemie (Buchs, Switzerland). DTNB (5,5-dithiobis-(2-nitrobenzoic acid), tris-HCl (tris-hydrochloride), and TBA (thiobarbituric acid) were purchased from Serva (Heidelberg, Germany). Standard solutions of cannabinoids (CBD, CBDA, CBG) and AlCl_3_ (>99.5%) were purchased from Sigma-Aldrich (St. Louis, MO, USA).

### 2.3. Preparation of Plant Samples

#### 2.3.1. Preparation of the Plant Extract for HPLC Analysis

Prior to preparing the extract, *Cannabis sativa* L. herbs were ground in an electric mill (D-47906 Clatronich, Kempen, Germany). The extraction procedure for CBD and CBG from hemp was followed by using ultrasound-assisted extraction with 96% ethanol, the material/solvent ratio being 1:10 and the extraction time being 10 min at room temperature. The extract was filtered through a 0.22 μm microfilter into a dark glass vial [20].

#### 2.3.2. Preparation of the Plant Extract used for Animal Studies

Prior to the preparation of the extract, *Cannabis sativa* L. herbs were ground in an electric mill (D-47906 Clatronic, Kempen, Germany). The powdered material (1 g) was placed in a 10 mL volumetric flask and extracted with 5 mL of 96% ethanol in an ultrasonic bath BioSonic UC100 (Maui, HI, USA) for 30 min. At the end of the ultrasonic extraction, the extract was cooled and diluted to an ethanol concentration of 10%. The extract was then filtered through a 0.22 μm microfilter into a dark glass vial.

### 2.4. High-Performance Liquid Chromatography (HPLC) Analysis

#### 2.4.1. HPLC Analysis for Identification of Cannabinoids

HPLC analysis was performed to detect CBD, CBDA, and CBG using a model Waters 2695 chromatography system (Waters Corporation, Milford, MA USA) equipped with a Waters 996 photodiode matrix detector (Waters Corporation, Milford, MA, USA). For separation, a 250 × 4.6 mm 5 µm ACE C18 column (Advanced Chromatography Technologies, Scotland) was kept in the external thermostat at a constant temperature of 40 °C. The mobile phase consisted of a mixture of acetonitrile (eluent A) and 20 mM ammonium formate, adjusted to pH 2.9 with formic acid (76:24). The separation of CBD, CBDA, and CBG was based on isocratic elution, the solvent flow rate was 1.5 mL/min, and the injection volume was 10 µL. The identification of chromatographic peaks was performed by matching the retention times of the analytes to reference standard compounds as well as UV absorption spectra limits of 210–400 nm.

#### 2.4.2. HPLC Post-Column Antioxidant Detection Analysis

After applying the HPLC-PDA detection system, the mobile phase containing the analytes was introduced into a reaction coil through a mixing tee, and reagents (DPPH solution or ABTS solution) were added (split ratio 1:1) at the same time by a Gilson pump 305 (Middleton, WI, USA). Reaction coils made of PEEK Teflon, 20 m for DPPH or 3 m for ABTS in length, 0.25 mm i.d., and 1.58 mm o.d. were used (Waters PCR module, Milford, CT, USA). The system was monitored as follows: the temperature range was set at 40 °C, and the flow rate was set at 1.0 mL/min of the DPPH reagent and 0.5 mL/min of the ABTS reagent. The reaction of the antioxidant compounds with the DPPH and ABTS reagents resulted in a color change that was detected using an additional Waters 2487 UV/VIS detector (Waters Corporation). Detection of ABTS and DPPH solution was at 650 nm and 520 nm, respectively. The post-column antioxidant activity of the extract compounds was assessed by comparing their activity to the standard, Trolox.

### 2.5. Experimental Animal Model

The study was performed on 4–6-week-old white male BALB/c laboratory mice weighing approximately 20–25 g. The extracts were administrated intragastrically to the mice via a stomach tube for 21 days. The administered volume of the sample was 10 mL/kg body weight. The mice had free access to food and water throughout the experiment. The mice were randomly assigned to 5 experimental groups regardless of their weight, each group consisting of 8 mice.

- The first group was injected (i.p.) with an aqueous solution of AlCl_3_ at a dose of 0.15 LD_50_ (7.5 mg/kg) Al^3+^ per kg body weight [18] per day for 21 days—control 1 group.
- The second group received the saline solution (NaCl 0.9%)—control 2 group.
- The third group received an appropriate amount of 10% ethanol solution for mouse weight—control 3 group.
- The fourth group received 1.6 mg CE (*Cannabis sativa* L. extract)/g/day (0.05 LD_50_).
- The fifth group received *Cannabis sativa* L. extract 20 min prior to an AlCl_3_ injection (7.5 mg AlCl_3_ plus 1.6 mg CE/g/day (0.05 LD_50_)).

The same markers were examined in all groups and were compared with the control 2 group. In addition, we evaluated how these parameters recovered compared to the aluminum group (control 1 group).

### 2.6. Blood Sample Preparation

The blood samples were centrifuged at 800× *g* for 10 min at 4 °C in the pre-heparinized tubes, and the plasma layers were removed. The erythrocytes were washed with phosphate-buffered saline (PBS, pH 7.4) three times and were kept on ice. The animals were culled in accordance with the rules defined by the European Convention for the Protection of Vertebrate Animals Used for Experimental and Other Purposes.

### 2.7. Preparation of Brain and Liver Homogenates

The preparation of brain and liver homogenates was evaluated according to the procedure described by Baranauskaite et al. [21]. Following cervical dislocation, the animal brain and liver were removed, washed, and immediately cooled on ice. The organs were carefully weighted and homogenized with 9 volumes (relative to organ weight) of cold 1.15% KCl solution, resulting in a 10% homogenate. The prepared homogenates were centrifugated at 15,000× *g* for 15 min. The separated post-mitochondrial supernatant was used for the measurement of enzymic activity in tissues.

### 2.8. Evaluation of GSH

GSH concentration in blood was evaluated according to the procedure described by Baranauskaite et.al [21]. During the evaluation, 200 µL of erythrocytes were mixed with 1.8 mL of deionized water and 2 mL of 0.6 M HClO_4_. The resulting mixture was centrifugated at 3000× *g* for 10 min. The upper aqueous layer was used for color reaction. Subsequently, 1 mL of the supernatant was mixed with 50 µL of DTNB (5,5-dithiobis-(2-nitrobenzoic acid)) stock solution (3.7 mg of DTNB in 1 mL of ethanol) and 3 mL of 0.4 M Tris-HCl (tris-hydrochloride) (pH 9.2). Light absorbance of the solution was determined via spectrophotometry at 412 nm.

### 2.9. Evaluation of MDA

One of the markers of lipid peroxidation is MDA. It can form a complex with thiobarbituric acid (TBA), which can be detected via spectrophotometry. The results are expressed as nmol/g of the wet weight of the tissue [22]. During the evaluation, 3 mL of 1% phosphoric acid were mixed with 0.5 mL of brain or liver homogenate and 1 mL of 0.6% TBA solution. The mixture was incubated for 45 min in a boiling water bath, was allowed to cool, and was mixed with 4 mL of n-butanol. The butanol phase was separated by centrifugation, and light absorbance of the supernatant was determined at 535 and 520 nm [23]. MDA concentration was evaluated in liver and brain samples.

### 2.10. CAT Activity Assay

The evaluation of CAT activity in mouse liver and brain homogenates was performed via the hydrogen peroxide and ammonium molybdate reaction, as the reaction product absorbs UV light at 410 nm. The activity of CAT was evaluated according to the procedure described in previous studies by Sadauskiene et al. [22]. The reaction mixture was prepared by mixing 100 µL of the organ homogenate with 5 mM Tris-HCl buffer (pH 7.4) and 30 mL of 0.08% H_2_O_2_ (buffer–substrate mixture), and the prepared mixture was incubated for 180 s at 37 °C. Subsequently, 2.0 mL of 4.5% ammonium molybdate were added to the mixture to stop the enzymic reaction. The absorbance of the molybdate complex and H_2_O_2_ was determined at 410 nm. The blank sample was prepared following the above-mentioned procedure (the buffer–substrate was incubated for 180 s at 37 °C degrees, and 100 µL of homogenate and ammonium molybdate were added). The obtained results were expressed as U/mg protein. One unit of CAT (U) decomposes 1 µmol of H_2_O_2_ per 1 min under these conditions.

### 2.11. Statistics

The obtained data were expressed as a mean ± standard error of mean (*n* = 8). The results were analyzed by applying the nonparametric Wilcoxon criterion for dependent samples and the unpaired Student *t*-test. Non-parametric tests were used because of a small study sample. The level of significance was taken as a value of *p* ≤ 0.05 (SPSS version 20.0, SPSS).

## 3. Results

**Figure 1:**
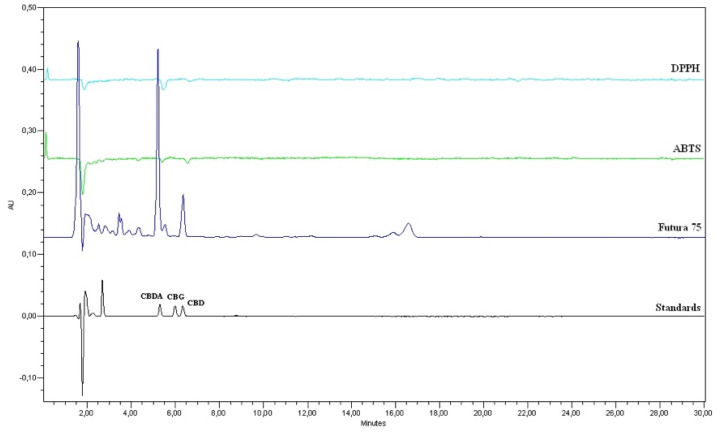
The identification and antioxidant activity of CBD and CBDA in *Cannabis sativa* L. extract determined by post-column HPLC.

Compounds with antioxidant activity (CBD and CBDA) were identified in samples of *Cannabis sativa* L. extract according to retention times and spectral compliance (Figure 1). The content of CBDA was found to be the highest, while CBG was not detected. The antioxidant activity of CBD and CBDA in *Cannabis sativa* L. extract was determined by using post-column HPLC. Comparing the areas of the peaks in the chromatogram, the HPLC post-column method with DPPH reagent is more sensitive than with ABTS reagent. Therefore, the quantitative evaluation of antioxidant activity was performed by the HPLC–DPPH post-column method. The evaluation showed that CBDA had the highest antioxidant activity (8.6 ± 0.1 µg/mL) than CBD (2.7 ± 0.01) (Figure 1).

**Figure 2:**
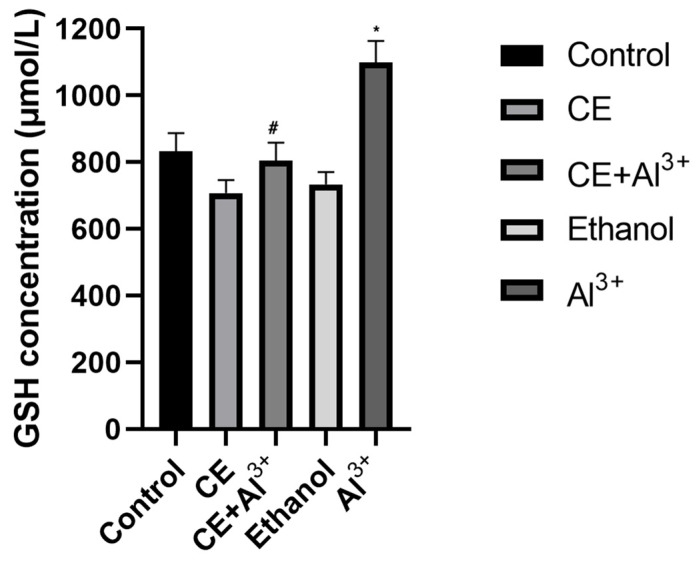
Influence of aluminum ions (Al^3+^), *Cannabis sativa* L. extract (CE), the combination of *Cannabis sativa* L. extract (CE) and aluminum ions (Al^3+^), ethanol, and control (physiological fluid) on the concentration of GSH in mouse erythrocytes (*n* = 8, * *p* < 0.05 vs. control, # *p* < 0.05 vs. Al^3+^).

*Evaluation of GSH*. The results revealed that the GSH level was lower (*p* > 0.05) in the blood of the mice that received *Cannabis sativa* L. extracts, but it was significantly (*p* < 0.05) higher (by 32.03%) in the blood of mice in the AlCl_3_ group compared with that in the vehicle control (control 2) group (Figure 2). In the mice treated with AlCl_3_ and *Cannabis sativa* L. extract, the amount of GSH was significantly lower (26.81%, *p* < 0.05) compared to that in the control 1 group, and decreased (*p* > 0.05) by 3.36% compared to its amount in the vehicle control (control 2) group (Figure 2).

**Figure 3:**
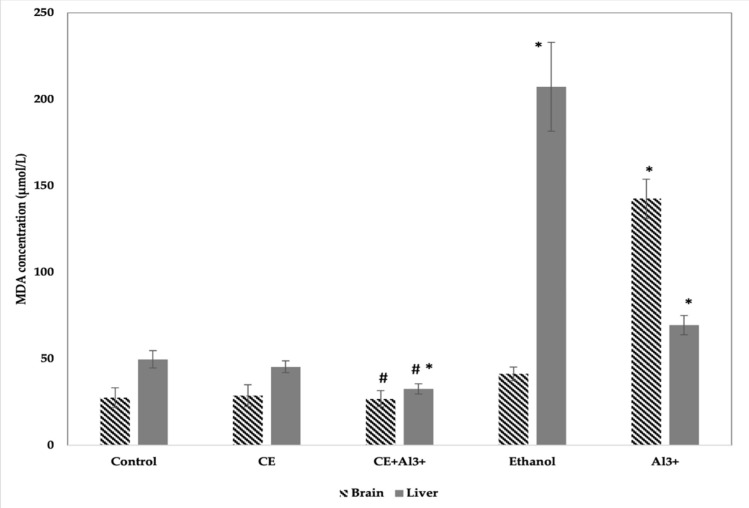
Influence of aluminum ions (Al^3+^), *Cannabis sativa* L. extract (CE), a combination of *Cannabis sativa* L. extract (CE) and aluminum ions (Al^3+^), ethanol, and control (physiological fluid) on the concentration of MDA in mouse brain and liver homogenates (*n* = 8, * *p* < 0.05 vs. control, # *p* < 0.05 vs. Al^3+^).

*Evaluation of MDA.* The data on the MDA in the tissues of control and experimental mice are presented in Figure 3. MDA levels in the brain were significantly greater (by 49.6%, *p* < 0.05) on ethanol supplementation (control 3) and on AlCl_3_ administration (by 416.64%) (control 1) than in the control mice group (control 2). In animals treated with *Cannabis sativa* L. extract, MDA concentration in the brain was only 4.28% (*p* > 0.05) higher than the control 2 group. On the other hand, in mice treated with a combination of AlCl_3_ and *Cannabis sativa* L. extract, the concentration of MDA was significantly lower (by 82.12%, *p* < 0.05) compared to that in the AlCl_3_ control group. The concentration of MDA in the liver of mice was significantly higher in the AlCl_3_ (control 1) and ethanol (control 3) groups than in the vehicle control group (control 2). *Cannabis sativa* L. extract caused no alterations in MDA concentration in the liver when compared with the respective findings in the control 2 group. Meanwhile, in the liver of mice treated with both AlCl_3_ and *Cannabis sativa* L. extract, the concentration of MDA was significantly lower (by 53.5%, *p* < 0.05) compared to that in the AlCl_3_ group.

**Figure 4:**
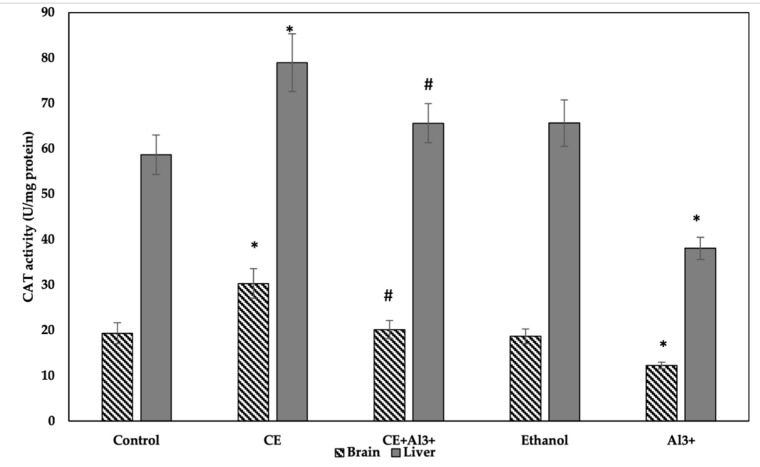
Influence of aluminum ions (Al^3+^), *Cannabis sativa* L. extract (CE), a combination of *Cannabis sativa* L. extract (CE) and aluminum ions (Al^3+^), ethanol, and control (physiological fluid) on the activity of catalase in mouse brain and liver homogenates (*n* = 8, * *p* < 0.05 vs. control, # *p* < 0.05 vs. Al^3+^).

*CAT Activity Assay.* The obtained results on the activity of CAT in the tissues of the control and the experimental groups are shown in Figure 4. The administration of AlCl_3_ (control 1) in the homogenates of the brain decreased the CAT activity by 36.84% (*p* < 0.05), whereas the administration of *Cannabis sativa* L. extract alone significantly increased it by 56.44%, compared to the activity in the vehicle control group (*p* < 0.05). The supplementation of 10% ethanol (control 3) and the administration of *Cannabis sativa* L. extract in combination with AlCl_3_ caused no alterations in CAT activity in the brain in comparison with the control group (control 2). However, the administration of *Cannabis sativa* L. extract in combination with AlCl_3_ significantly increased the activity of CAT in the brain compared with that in the control 1 group, and it reached the same level as in the vehicle control group (control 2). Changes in the activity of CAT in the liver of the mice followed the same pattern: the administration of AlCl_3_ (control 1) significantly decreased (by 35.13%, *p* < 0.05) the activity of CAT, the administration of *Cannabis sativa* L. extract alone significantly (by 34.53%, *p* < 0.05) increased it, and supplementations with 10% ethanol (control 3) caused no alterations in CAT activity in the liver in comparison with that in the control group (control 2). The administration of *Cannabis sativa* L. extract in combination with AlCl_3_ significantly increased CAT activity in the liver in comparison with that in the AlCl_3_ control group.

## 4. Discussion

*Cannabis sativa* L. produces multiple chemical compounds of different biogenetic classes [15], and many studies have demonstrated the antioxidant activity of *Cannabis* in vitro [19,22]. The phenolic compounds are mainly responsible for the antioxidant activity, while hemp seeds due to their active compounds, have health benefits such as lowering the cholesterol level in the blood or alleviating constipation and are also useful in the treatment of cardiovascular diseases [11,23]. Cannabinoids protect against oxidative stress in the cell [13], and cannabinoid-like CBD has anxiolytic, antipsychotic, and antidepressant properties [24]. However, in fresh *Cannabis sativa* L. plants, CBD exists primarily in the carboxylated form, cannabidiolic acid [25].

In this study, the antioxidant activity of CBDA in vitro was detected, and this finding is in line with literature data suggesting that this cannabinoid has antioxidant properties [14]. However, this study was intended to investigate the effect of the whole extract of *Cannabis sativa* L. on oxidative stress markers. The antioxidant activity of CBDA was found to be low in vitro, and thus it could be concluded that other compounds (flavonoids or/and terpenoids) in the extract also have an effect on oxidative stress markers, which corroborates literature data claiming that flavonoids and terpenoids have a wide therapeutic window [15].

Considering the fact that *Cannabis* extract also contains alcohol, we evaluated the effect of ethanol on the antiradical activity in the brain and liver of experimental mice. The results of the evaluation were similar to those obtained in our previous study [22].

According to Kumar et al. and Singh et al., Al^3+^ has a tendency to accumulate in all regions of rat brain following chronic exposure and exhibits significant neurotoxicity as assessed by behavioral, biochemical, and histopathological estimations [26]. However, the researchers found that brain accumulates lower amounts of Al^3+^ than do liver, spleen, lungs, or many other tissues, but accumulates higher amounts in comparison with blood plasma [26]. Much research has been carried out to conduct the correlation between Al^3+^ accumulation and oxidative damage in the body tissues [27,28]. The obtained results could be explained by the fact that Al^3+^ is known to act as a prooxidant and to be capable of inducing oxidative stress by disturbing the activity of antioxidant enzymes such as SOD, CAT, and glutathione reductase, and by depleting sulfhydryl [29]. In our study, no symptoms of subacute administration of AlCl_3_ for 21 days were observed, but it significantly increased GSH levels in blood and MDA levels in the liver and brain of the experimental mice and decreased CAT activity in the brain and liver of the mice. This confirms that Al^3+^ intake produces oxidative stress, and these data are similar to those obtained by other researchers [30].

We observed no significant changes in GSH levels in blood or MDA levels in the brain and liver of experimental mice after exposure to *Cannabis sativa* L. extract. However, it significantly decreased these concentrations after AlCl_3_-induced oxidative stress and reached the same concentration as in the vehicle group (control 2).

CAT activity is considered to be a sensitive biomarker of oxidative stress [31]. A decrease in catalase activity in cells indicates a state of oxidative stress in the cell. In our study, treatment with *Cannabis sativa* L. extract alone showed a significant difference in CAT activity in the liver and brain of the experimental mice compared to that in the control group 2 (Figure 4). As Al^3+^ can decrease the activity of enzymes related to cell antioxidant defense, such as CAT, in the liver and brain [30], *Cannabis sativa* L. extract significantly increased the activity of this antioxidant enzyme after AlCl_3_-induced oxidative stress, and it reached similar concentrations as in the control 2 group. *Cannabis sativa* L. contains chemical compounds of various classes, e.g., terpenes, flavonoids, cannabinoids, amino acids, fatty acids, and others [32]. According to literature, besides protein and fatty acids, phenylpropanamides are widely studied coactive biological ingredients. More than 20 kinds have been extracted from hemp seed, and some showed good antioxidant and anti-neuroinflammatory properties [33]. Currently, the most researched secondary metabolites of hemp seeds are lignanamides [34], which have demonstrated a powerful antioxidant and free radical scavenging effect using a chemical assay [35].

Our results also suggest that *Cannabis* extract may regulate oxidative stress as it has a significant role in increasing the activity of CAT in plasma. This is consistent with the results of other studies showing the ability of hemp seed peptides to increase CAT activity in plasma [35].

Our results demonstrated that *Cannabis sativa* L. extract significantly altered GSH and MDA concentrations as well as CAT activity associated with AlCl_3_-induced toxicity. The negative effect of Al^3+^ on oxidative parameters has been shown previously by Hammoud et al. However, contrary to our data, they found an increase in MDA concentration along with a depletion of GSH in the plasma of Al-intoxicated rats [27]. Candan and Tuzmen quantified MDA levels in rat brain under the action of lead, aluminum, and phenolic antioxidants [26]. It is already known that Al^3+^ accumulates in different organs, increases ROS generation, can enhance lipid peroxidation in liposomes, alters membrane fluidity, and induces free radical-mediated cytotoxicity [36]. Elevated MDA levels and decreased CAT activity in AlCl_3_-treated rats were also found in a study by Bouasla et al. [37], suggesting that these changes may be related to peroxidative damage to biological membranes caused by increased reactive Fe^2+^ levels and the inactivation of enzymes involved in antioxidant defense [37]. Our previous study proved the antioxidant activity of the *Cannabis sativa* L. extract in vitro, which was related to the total amount of polyphenols [21] due to their ability to chelate metal ions and scavenge reactive oxygen species [27]. The present study showed the ability of this extract to stimulate the antioxidant defense system in vivo.

## 5. Conclusions

In this study, we determined antioxidant activity of CBD and CBDA, and CBDA had a higher antioxidant activity in vitro compared to CBD. This in vivo study showed a decrease in tissue (liver and brain) MDA levels and an increase in the antioxidant enzyme (CAT) activity in animals that were administered *Cannabis sativa* L. (var. Futura 75) extract in combination with AlCl_3_, in comparison to the group that was administered *Cannabis* extract alone. By increasing the activities of antioxidant enzymes, this extract reduces the levels of free radicals and ROS and increases the production of molecules capable of protecting against oxidative stress. This is important to expand knowledge about the effects of *Cannabis* in the prevention of chronic diseases and health problems. Due to the results of low antioxidant activity of CBDA and CBD, it is appropriate to extend studies to determine which antioxidant components (terpenes, phenols, etc.) have the greatest influence on oxidative stress markers in vivo.

## Data Availability

Not applicable.
